# Supplementary material for: Content-rich biological network constructed by mining PubMed abstracts
Source: BMC Bioinformatics. 2004 Oct 8;5:147. doi: 10.1186/1471-2105-5-147 (PMC528731; doi:10.1186/1471-2105-5-147)
Supplement: Additional File 2 — The original results of the above study (non-essential files are deleted to keep the file size under the limit set by BMC bioinformatics). [file 1471-2105-5-147-S2.bz2 › chilibotAdditionalFile2/dip10/575ID9204896E10943/html/ANGPT1_TEK.html]

 


 **ANGPT1** and **TEK** 
  
Found 178 abstracts in PubMed, retrieved 10.  
 

 What does Google say? 
 PDF only 
| .edu only 

---

**Interactive relationship** (e.g. stimulation, inhibition, etc)

**Stimulatory relationship**- Ang 1  [ **ANGPT1** ]  activates the endothelial cell specific tyrosine kinase receptor Tie 2  [ **TEK** ] , which in turn leads to enhanced endothelial cell survival and stabilization.  Ref: 12810673 Cancer Res, 2003
- Using a combination of immunohistochemistry, laser capture microdissection and real time quantitative reverse transcription PCR, we showed that tumor derived VEGF significantly up regulated the expression of Ang  [ **ANGPT1** ]  2 in host stroma endothelial cells, resulting in markedly increased Ang  [ **ANGPT1** ]  2 Tie 2  [ **TEK** ]  mRNA copy number ratio in vivo.  Ref: 12810677 Cancer Res, 2003
**Neutral relationship**- Recent studies have implicated the Tie2  [ **TEK** ]  tyrosine kinase receptor and its main ligands angiopoietin 1  [ **ANGPT1** ]  Ang 1  [ **ANGPT1** ]  and angiopoietin 2 Ang  [ **ANGPT1** ]  2 as crucial regulators of mural cell recruitment during angiogenesis.  Ref: 12876214 J Cell SciJ Cell Sci, 2003
- These data provide comprehensive evidence that VEGFR 1 and 2, and angiopoietin 1  [ **ANGPT1** ] , angiopoietin 2 and Tie 2  [ **TEK** ]  interactions may be involved in the preparation of endometrium for implantation, remodelling of the maternal vasculature and trophoblast invasion during the peri implantation period in this primate species.  Ref: 12887279 Reproduction, 2003
- Two of the Angs, Ang 1  [ **ANGPT1** ]  and Ang  [ **ANGPT1** ]  4, activate the Tie2  [ **TEK** ]  receptor.  Ref: 12783598 Expert Opin Investig Drugs, 2003
- 1 Angiopoietin 1  [ **ANGPT1** ]  Ang1  [ **ANGPT1** ]  is an angiogenic growth factor that binds to the Tie2  [ **TEK** ]  receptor on vascular endothelium, promoting blood vessel maturation and integrity.  Ref: 12770938 Br J Pharmacol, 2003
- Tie 2  [ **TEK** ]  dependent activation of RhoA and Rac1 participates in endothelial cell motility triggered by angiopoietin 1  [ **ANGPT1** ] .  Ref: 12816861 Blood< MedlineTA>Blood, 2003
- The angiopoietin Ang  [ **ANGPT1** ]  family of growth factors includes four members, all of which bind to the endothelial receptor tyrosine kinase Tie2  [ **TEK** ] .  Ref: 12783598 Expert Opin Investig Drugs, 2003
- ... Ang  [ **ANGPT1** ]  2 and Ang  [ **ANGPT1** ]  3 inhibit Ang 1  [ **ANGPT1** ]  induced Tie2  [ **TEK** ]  phosphorylation.  Ref: 12783598 Expert Opin Investig Drugs, 2003

**Non-interactive relationship** (e.g. studied together, co-existance, homology, etc.)

- Immunoprecipitation showed phosphorylation of MPC Tie2  [ **TEK** ]  on tyrosine residues upon stimulation with Ang 1  [ **ANGPT1** ]  or Ang  [ **ANGPT1** ]  2.  Ref: 12876214 J Cell SciJ Cell Sci, 2003
- Using RT PCR and immunohistochemistry we demonstrate that testicular vascular endothelial growth factor A VEGF A, ang 1  [ **ANGPT1** ] , ang  [ **ANGPT1** ]  2 and the tie 2  [ **TEK** ]  angiopoietin receptor are expressed in the testis.  Ref: 12773423 Biol Reprod< MedlineTA>Biol Reprod, 2003
- mRNAs for VEGF, VEGFR 1 and VEGFR 2, angiopoietin 1  [ **ANGPT1** ] , angiopoietin 2 and their receptor Tie 2  [ **TEK** ]  were localized and quantified by in situ hybridization.  Ref: 12887279 Reproduction, 2003
- Angiopoietin 1  [ **ANGPT1** ]  Ang1  [ **ANGPT1** ]  and its receptor, Tie2  [ **TEK** ] , play critical roles in blood vessel formation.  Ref: 12890486 Biochem Biophys Res Commun, 2003
- However, the underlying mechanism for Ang1  [ **ANGPT1** ]  Tie2  [ **TEK** ]  signaling is not fully understood.  Ref: 12890486 Biochem Biophys Res Commun, 2003
- Polyclonal antibodies specific for Ang1  [ **ANGPT1** ] , Ang2, Tie2  [ **TEK** ]  and VEGF were used for immunostaining.  Ref: 12815197 Ophthalmic Res, 2003
- 0.01 but not between VEGF and Ang 1  [ **ANGPT1** ]  or Tie 2  [ **TEK** ]  in human ovarian cancer specimens.  Ref: 12810677 Cancer Res, 2003
- 2 PLD is a new mediator of Ang1  [ **ANGPT1** ]  Tie2  [ **TEK** ]  induced signaling pathway, and it participates in MAPK activation and endothelial cell migration.  Ref: 12890486 Biochem Biophys Res Commun, 2003
- Localization of Tie2  [ **TEK** ]  and phospholipase D in endothelial caveolae is involved in angiopoietin 1  [ **ANGPT1** ]  induced MEK ERK phosphorylation and migration in endothelial cells.  Ref: 12890486 Biochem Biophys Res Commun, 2003
